# Sealing agent reduces formation of single and dual-species biofilms of *Candida albicans* and *Enterococcus faecalis* on screw joints at the abutment/implant interface

**DOI:** 10.1371/journal.pone.0223148

**Published:** 2019-10-22

**Authors:** Cecília Alves de Sousa, Jadison Junio Conforte, Karina Sampaio Caiaffa, Cristiane Duque, Wirley Gonçalves Assunção

**Affiliations:** 1 Department of Dental Materials and Prosthodontic, São Paulo State University (UNESP), School of Dentistry, Araçatuba, São Paulo, Brazil; 2 Department of Pediatric Dentistry and Public Health, São Paulo State University (UNESP), School of Dentistry, Araçatuba, São Paulo, Brazil; Louisiana State University, UNITED STATES

## Abstract

The aim of this research was to evaluate the efficacy of a commercial sealing agent at the abutment/implant interface against microleakage of single and dual-species biofilms of *Candida albicans* and *Enterococcus faecalis* into external hexagon (EH) and Morse taper (MT) prosthetic connections. A total of 216 samples of implants and their abutments were tested. Six groups (n = 36) were evaluated based on biofilm and period of incubation (7 and 14 days). The implant connections EH and MT (n = 18) were divided according to the use of the material (n = 9) (EH-T and MT-T: with the sealing agent; EH-C and MT-C: control). The biofilms were analyzed by microbial counting (CFU/mL) and SEM analysis and photographs of the material in the screw joints were also taken. Data were analyzed by Student t test, two-way ANOVA and Bonferroni test. For the single-species biofilms, there was a significant reduction in the growth of *E*. *faecalis* when compared MT-C and MT-T or EH-C and EH-T at 7 and 14 days. The same was observed for *C*. *albicans* biofilms. For dual-species biofilms of *E*. *faecalis* and *C*. *albicans*, the sealing agent was more effective in preventing microbial infiltration into the MT connection at 14 days, while microbial infiltration did not occur into EH connections even in absence of the sealing agent for both periods of evaluation. Overall, these data suggest that the presence of the sealing agent reduces or eliminates the microleakage of *E*. *faecalis* and *C*. *albicans* biofilms into the implants regardless of the period of incubation.

## Introduction

Numerous studies have reported high success rates with regard to dental implant treatment. However, several complications that may lead to implant loss can occur during and after the period of osseointegration. [1] One of the reasons for the failure of implants is the increased prevalence of peri-implant infections, which are multifactorial and immune-mediated inflammatory diseases that affect the supporting tissues surrounding the implanted area, leading to loss of the adjacent bone. [2]

Despite the histological and structural differences between the teeth and the implants there are clinical similarities with regard to the diseases that affect the periodontal tissue. [3, 4] Thus, previous history of periodontal diseases may also be considered as a risk factor for patients receiving dental implants. Schou (2008) has previously discussed an increased incidence of peri-implantitis and bone loss due to periodontal disease in patients with implants.

These facts suggest that microorganisms that cause periodontal disease can migrate and colonize peri-implant sites. [5] Thus, the development of the subgingival microbiota is directly related to the influence of the supragingival microbiota. Studies have suggested that even after tooth loss, in completely edentulous subjects, the species of microorganisms related to periodontal disease were present in the oral cavity, soft tissue, and the alveolar bone. [6, 7]

*Enterococcus faecalis* (*E*. *faecalis*) is one of the microorganisms found in bone tissues in cases of peri-implantitis and periodontitis. [8, 9] It is classified as gram-positive, facultative-anaerobic, commensal cocci, inhabiting the gastrointestinal tract. *E*. *faecalis* can exist without causing signs or symptoms of disease, in a opportunistic way, inside the bone; however, an osteotomy in an edentulous site activates the microorganism. In this way, when a dental implant is placed, it provides for bacterial colonization, forming a biofilm on the dental implant surface. [10]

*Candida* species, a genus of yeast, are also frequently associated with biofilm formation in dental implants [11]. They are commensal fungi, which can be found in biofilms of peri-implant areas and usually infect only immunocompromised hosts. In addition, *Candida* cells can bind to bacteria that have already colonized a foreign body, thus becoming a part of the formed bacterial biofilm. [11]

In case of dental implants, in addition to colonizing their external surface, microorganisms can also be established at the interface of the implant and the prosthesis connection [12], specifically in the micro-gap formed between the implant and the prosthetic abutment. Microorganisms colonizing the outer surface can be eliminated by the host defense mechanisms; however, the microorganisms that internally colonize the implants and the interfaces of the parts can persist within for long periods, causing unpleasant odor and taste, infections, and tissue damage. [13]

*In-vivo* and *in-vitro* studies have demonstrated the presence of viable microorganisms in the internal parts of the implants and infiltration of fluids and microorganisms throughout their internal space, which may lead to contamination of the tissues near the installed dental implants. [14, 15]

In an attempt to test the effectiveness of materials that would seal this interface, Duarte et al. (2006), suggested the application of chlorhexidine varnish and silicone sealant; however, they did not show an effective sealing for more than 35-days, demonstrating their lack of ability to seal the abutment-implant interface for prolonged periods. Other materials have been extensively studied, such as gutta-percha [16], Gap-Seal gel [17], polytetrafluoroethylene based materials, and composite resin [18]. Although, these materials present favorable results, they are also not durable and work against microbial infiltration only for a short period of time.

In this context, there has been an interest shown towards using sealing materials at the abutment-implant interface in order to minimize or even prevent microbial penetration. One such material that was evaluated by Seloto et al (2018) showed promising results for its performance in maintaining the preload of screwed abutment-implant joints; it is often used in mechanics and classified as "chemical locking.” [19] They used resins with mono components, without solvents, that polymerize at room temperature in the absence of oxygen, when trapped between the parts. Such conditions prevented the loosening of nuts and bolts caused by vibrations, by filling the voids between the threads, molding to the roughness, and forming a unique body [19].

Similarly, this study evaluated the sealing ability of a mono component, anaerobic, and thixotropic material with low resistance to disassembly, commonly being used industrially to adjust screws. This methacrylate-based product is suitable for locking and sealing threaded surfaces, the dismantling of which requires conventional hand tools. The product curing, when confined between metal surfaces in the absence of air, prevents loosening and leakage caused by impact and vibration. It is especially suitable for applications on less active surfaces, such as stainless steel and treated surfaces.

The aim of this research was to evaluate the efficacy of a sealing agent at the abutment-implant interface by preventing infiltration and formation of single and dual-species biofilms of *C*. *albicans* and *E*. *faecalis* in two prosthetic dental implant connections (external and internal hexagonal Morse taper), at different incubation periods (7 and 14 days). The null hypothesis established was that there was no significant difference in the microbial infiltration and biofilm formation by *C*. *albicans* and *E*. *faecalis* when inoculated alone or together, regardless of the type of connection and the period of incubation.

## Materials and methods

### Study groups and sealing agent

**Fig 1:**
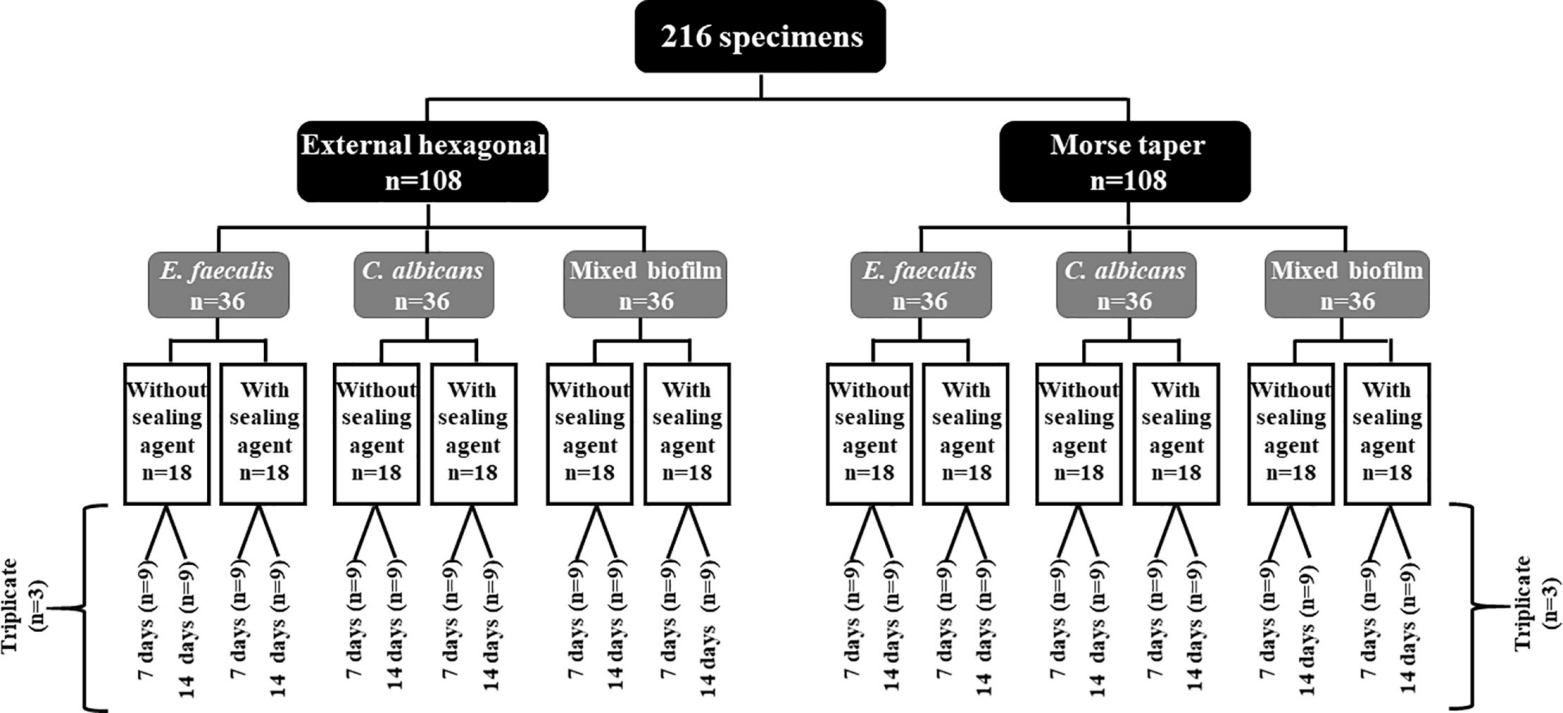
Organization chart illustrating the division of the groups.

A total of 216 titanium alloys implants of 4 mm diameter were connected to their respective UCLA-type prosthetic abutments and were retained by the appropriate retaining screws, (DSP Biomedical, Campo Largo, Paraná, Brazil). Two types of implant connections, external hexagon (EH) (n = 108) and Morse taper (MT) (n = 108) were evaluated. Three types of biofilms were analyzed: *C*. *albicans* single biofilms (n = 36), *E*. *faecalis* (n = 36) single biofilms, and *C*. *albicans* and *E*. *faecalis* mixed biofilms (n = 36). For each type of biofilm, the sets were subdivided into two groups according to the use of the sealing agent: test groups (EH with sealing agent [EH-T] [n = 18]; MT with sealing agent [MT-T] [n = 18]), and control groups (EH without sealing agent [EH-C] [n = 18]; MT without sealing agent [MT-C] [n = 18]). The biofilms formed in these sets were evaluated for two time periods, 7 and 14 days (n = 9). For biofilm analysis, three experiments were carried out on independent occasions using three samples from each group and were performed in triplicates. (Fig 1)

### Bacterial strains and growth conditions

Two microorganisms were used: *C*. *albicans* (ATCC 90028) and *E*. *faecalis* (ATCC 29212) [20]. The microorganisms were reactivated and cultivated separately in 5 mL Brain Heart Infusion (BHI) broth culture medium (Difco, Kansas City, MO, USA) to obtain the growth curve of the microorganisms, determined by their Optical Density (OD) values [21]. After reaching the log phase of growth (OD = 0.3 for *C*. *albicans* and OD = 0.5 for *E*. *faecalis*), microbial cultures were adjusted to 1.5 × 10^8^ cells/mL, by dilution in the BHI broth.

### Biofilm and collection assay for microbiological processing

#### Contamination control of the experiment

The experiments were carried out in a biosafety cabinet (Veco–Campinas–SP, Brazil). The sealing agent was sterilized by ultraviolet light from the cabinet for 20 minutes. Two calibrated operators performed the experiments. The first operator manipulated only the sterile materials with sterile gloves and scrubs. The second operator used conventional gloves and had contact with the rest of the instruments that were not sterile, thus avoiding the contact with sterile materials in order to maintain the aseptic conditions.

#### Bacterial contamination—Control group

The experiments were conducted with the first operator keeping the implant in a vertical position by means of hemostatic tweezers [22] and the second operator inserting 2 μL of sterile BHI broth culture medium into the implants. After that, the first operator positioned the respective abutments, threaded their bolts, and applied the torque (20 Ncm for MT implants and 30 Ncm for EH implants). Prosthetic abutments were closed with sterile cotton and temporary restorative material (Obtur—Maquira e Produtos Odontológicos S.A–Maringá–PR, Brazil) to prevent their opening and posterior infiltration into the implant.

#### Bacterial contamination—Test group

**Fig 2:**
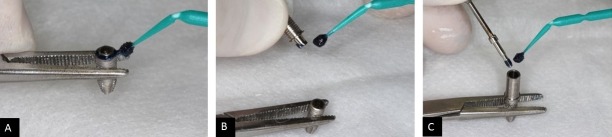
A. Application of the material to the hexagonal portion of the implant HE; B. Application in the indexing portion of the prosthetic abutment CM; C. Application of the material to the threads of the retaining screw.

After 2 μL sterile BHI broth medium to be inserted into the implants, a thin layer of the sealing agent (previously sterilized with ultraviolet light for 20 minutes) was applied to the implant platform, to the abutment, and to the threads of the retaining screw, with a sterile micro-brush (Fig 2). Then, the respective abutments were positioned on the implants, their bolts were threaded, and the torque was applied. The same procedures adopted for the control group were conducted for the test group considering torque parameters and prosthetic abutments closure.

Specimens from both groups (test and control), were inserted into tubes containing 5 mL and 1.5 x 10^8^ of either one of the bacterial cultures: *E*. *faecalis*, *C*. *albicans*, or a mixed culture containing both *C*. *albicans* and *E*. *faecalis* (2.5 mL of each microbial culture). The tubes were incubated at 37°C in 5% CO_2_ atmosphere for 7 and 14 days. Every 2 days, the culture medium was renewed to the same original volume in order to avoid microbial starvation.

#### Material collection and microbial counting

After 7 and 14 days of incubation, the culture medium inside the implants was collected. The specimens were removed from the tubes and rubbed in gauze soaked with 70% alcohol for external decontamination. The assembly was stabilized in an upright position and the prosthetic abutment screw was removed. Subsequently, 10 μL of saline solution (0.9% NaCl) was carefully inserted into implants and the walls of the implants were scraped with the aid of a 10μL micropipette tip. The solution was resuspended in order to recover the culture medium that had been previously placed and microorganisms that could be trapped between the threads [23]. Next, 5 μL of this solution was serially diluted, plated in three different media, according to the biofilm type and incubated at 37°C in 5% CO_2_ atmosphere for 48 hours. For single species biofilms, BHI agar media was used for total *E*. *faecalis* counting and Sabouraud Dextrose agar was used for total *C*. *albicans* counting. For dual-species biofilms, BHI agar containing amphotericin (20 µg/mL) was used for *E*. *faecalis* counting [24] and Sabouraud Dextrose Agar containing chloramphenicol (40 µg/mL) was used for *C*. *albicans* counting [25].

### Biofilm analysis by scanning electron microscopy (SEM)

One representative specimen from each group (CM-C, CM-T, EH-C, EH-T after 7 and 14 days of single or dual-species biofilms) was analyzed by SEM. The abutment and implant were analyzed separately and the connecting region of both the pieces was also analyzed after positioning the specimen in a vertical position. The specimens were pre-fixed in serial dilutions of alcohol (70% for 5 minutes, 90% for 5 minutes, and 100% for 20 minutes), dried under aseptic conditions in a biosafety cabinet [24], and subjected to metallization with gold blasting. The analysis was performed at two distinct sites of the specimens, at 1000× magnification using a scanning electron microscope.

### Visual analysis of bolted joints and sealing agent inside

The specimens of each test group were embedded in a colorless, self-curing acrylic resin (VIPI Flash, VIPI Produtos Odontológicos, Pirassununga–SP—Brazil) by means of an electric filler (Arotec PRS-30S –Arotec Indústria e Comércio–Cotia—SP, Brazil). They were then positioned and subjected to coronal cutting using a precision cutter (Isomet 5000, Buehler–Illinois, EUA). The parts obtained were photographed using a digital camera with a 100 mm macro lens (Canon EOS 70D –Ota–Toquio, Japan).

### Statistical analysis

Statistical analysis was performed using IBM SPSS Statistics version 17.1 (Statistical Package for the Social Sciences, IBM Corporation, New York, EUA). The Kolmogorov-Smirnov homogeneity test was applied to data and normal distribution was observed. In order to compare the mean CFU/mL count of the single-species biofilm assays of *E*. *faecalis* and *C*. *albicans*, the two-way ANOVA test was carried out followed by the Bonferroni test. A level of significance of 5% was considered statistically significant. For dual-species biofilms, student *t*-test was used. The influence of the sealing agent and the incubation period (7 and 14 days) were analyzed. The number of CFU/mL was logarithmically transformed into log (CFU + 1) due to the high microbial counts. The “+1” was added in order to factor in the zero values that were found in this study (log 1 = 0).

## Results

**Fig 3:**
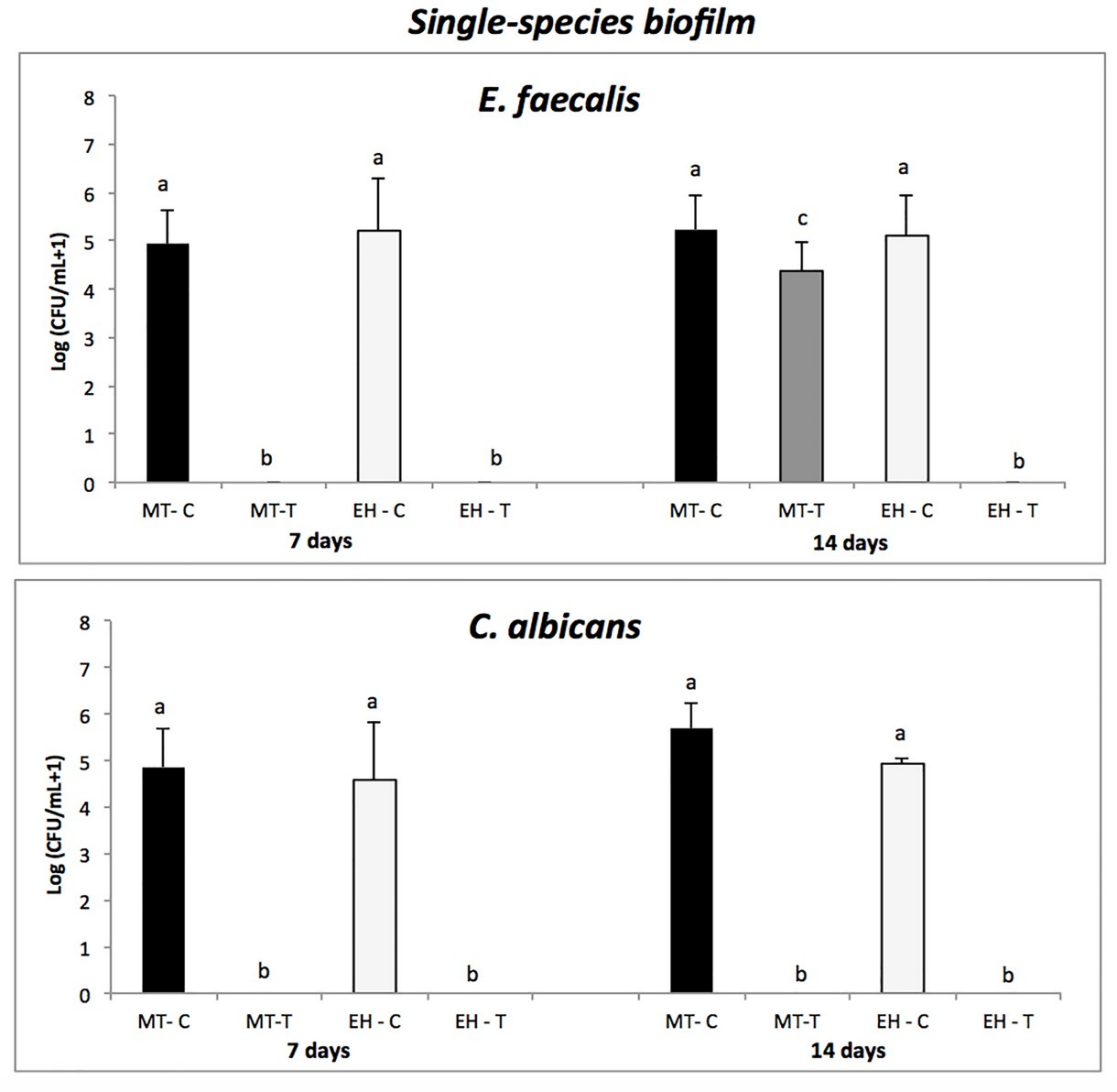
Single-species biofilms. A. *E*. *faecalis* biofilms—Mean (standard deviation) of *E*. *faecalis* counts (in logarithmic scale) recovered from implants (MT and EH) of control (C) and test (T) groups. B. *Candida albicans* biofilms. Mean (standard deviation) of *C*. *albicans* counts (in logarithmic scale) recovered from implants (MC and EH) of control (C) and test (T) groups. Different lowercase letters show statistical difference between each group and the time of evaluation, according to ANOVA/Bonferroni tests, considering *p<* 0.05 as statistically significant.

Fig 3 shows the counting of *E*. *faecalis* or *C*. *albicans* recovered from implants after 7 and 14 days in single-species biofilms.

Fig 3A shows total count (log (CFU/mL + 1)) of *E*. *faecalis* recovered from implants of control and test groups after 7 and 14 days of incubation. After 7 days of incubation, a statistically significant difference was observed in the growth of *E*. *faecalis* between the Morse taper control and test groups (MT-C vs. MT-T) (*p* = 0), with bacterial growth detected only in the control group (without sealing agent). The same performance was observed for external hexagonal connection (EH-C vs. EH-T), with bacterial growth observed only in the control group. Intergroup analysis showed a significant difference in the bacterial growth between the test groups of Morse taper connection at 7 and 14 days (MT-T 7 days vs. MT-T 14 days). After 14 days of incubation, the intragroup analysis showed a statistically significant difference (*p* = 0.04) in the bacterial growth in the conical connection groups (MT-C vs. MT-T), with the highest bacterial count in the MT-C group (5.22 ± 0.71). The hexagonal connection groups (EH-C vs. EH-T) showed a statistically significant difference in the bacterial growth in the two groups with growth detected only in the EH-C group (5.10 ± 0.83). For the control groups of both connections (MT-C vs. EH-C), there was no statistically significant difference in the bacterial growth, while the bacterial growth in the test groups (MT-T and EH-T) differed significantly from each other (*p* = 0.02), and the EH-T group did not present any bacterial growth at all.

Fig 3B shows total count (log (CFU/mL + 1)) of *C*. *albicans* recovered from implants of control and test groups after 7 and 14 days of incubation. After 7 days of incubation, a statistically significant difference was observed in the growth of *C*. *albicans* between the test and control conical connection groups (MT-C vs. MT-T) as well as between the external hexagonal groups (*p* = 0) (EH-C vs. EH-T), with microbial detection only in the control groups (without sealing agent). However, there were no significant differences (*p* = 1) in the bacterial growth between the control and test groups (MT-C vs. EH-C and MT-T vs. EH-T). After 14 days of incubation, the intergroup analysis showed a statistically significant difference (*p* = 0) between the conical connection groups (MT-C and MT-T). On the analysis of the hexagonal connection group (EH-C and EH-T), a statistically significant difference (*p* = 0) was observed in the bacterial growth between groups, with growth observed only in the EH-C group (4.93 ± 0.09). Considering the intergroup analysis (MT-C vs. EH-C and MT-T vs. EH-T), the control and test groups did not present a statistically significant difference between themselves (*p* = 1). There was no statistical difference between similar connections as a function of time (*p* = 1).

**Fig 4:**
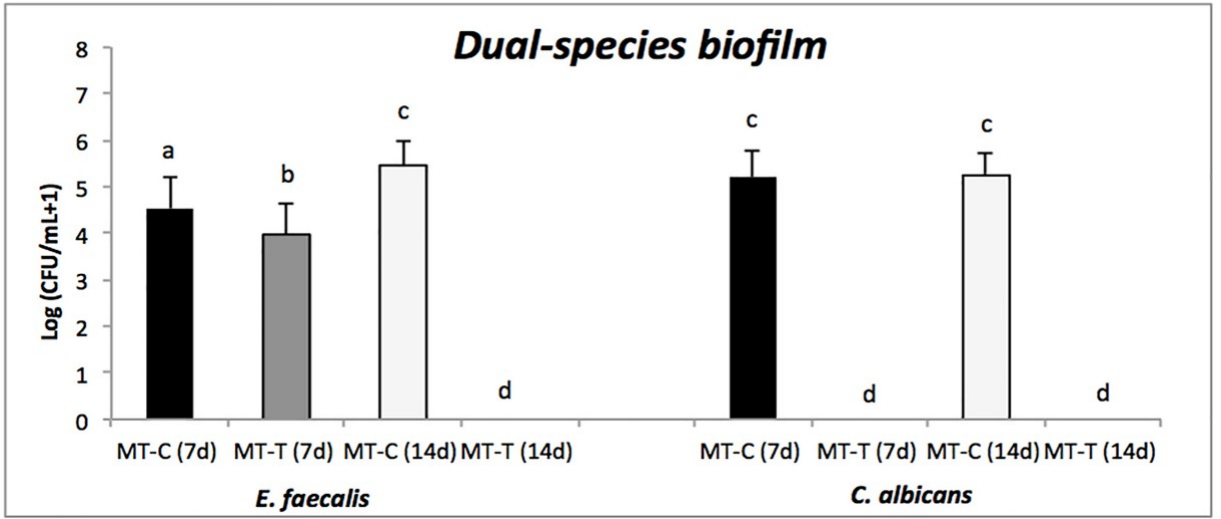
Dual-species biofilms. Mean (standard deviation) of *E*. *faecalis* and *C*. *albicans* counts (in logarithmic scale) recovered from implants (MT and EH) of control (C) and test (T) groups. Different lowercase letters show statistical difference between each group and time of evaluation, according to ANOVA/Bonferroni tests, considering *p<* 0.05 as statistically significant.

Fig 4 shows total counts (log (CFU/mL + 1)) of *E*. *faecalis* and *C*. *albicans* in dual-species biofilms recovered from Morse taper implants connections, after 7 and 14 days of incubation.

No microbial growth was observed in the external hexagonal connections, irrespective of the microorganism and the incubation period. Thus, there was a statistically significant difference between the control and test group connections across periods of time regardless of the microorganism. After 7 days of incubation, significant difference was observed in *E*. *faecalis* counts in the MT-T group in comparison to its control (MT-C). After 14 days, there was no bacterial growth in the MT-T group, only in the MT-T group. Moreover, upon comparing the two periods of time, 7 and 14 days, there was a significant difference in the bacterial growth between the both groups MT-C and MT-T, across the time period. For *C*. *albicans* in the dual-species biofilms, growth was detected only in the control group, regardless of the time of evaluation. We also observed that there was no significant difference in the bacterial growth between the control groups (MT-C 7 days and MT-C 14 days).

### Analysis of biofilms by scanning electron microscopy (SEM)

**Fig 5:**
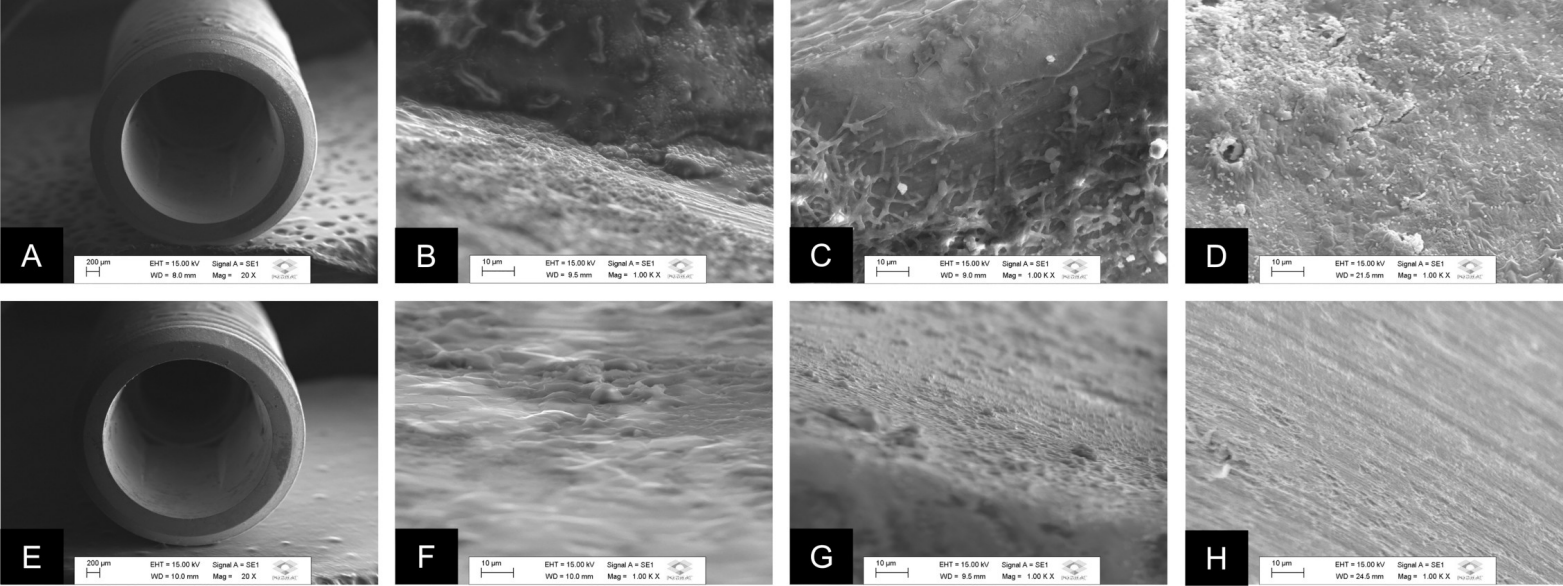
Representative micrographs of the MT specimens after 7 days of incubation with *E*. *faecalis* and *C*. *albicans*, in single or dual-species biofilms, obtained by SEM. A. External region of the CM-C implant; B. CM-C with *E*. *faecalis* in single-species biofilm formed in the internal region of the implant; C. CM-C with *C*. *albicans* in single-species biofilm formed in the internal region of the implant; D. CM-C with dual-species biofilm and extracellular matrix of *E*. *faecalis* and *C*. *albicans* formed in the internal region of the implant; E. External region of CM-T implant with the presence of the sealing agent at the platform; F. CM-T implant with absence of *E*. *faecalis* in single-species biofilm and presence of the sealing agent in the internal region of the implant; G. CM-T with absence of *C*. *albicans* biofilm in single-species biofilm and presence of the sealing agent in the internal region of the implant; H. CM-T with the absence of *E*. *faecalis* and *C*. *albicans* in dual-species biofilm.

**Fig 6:**
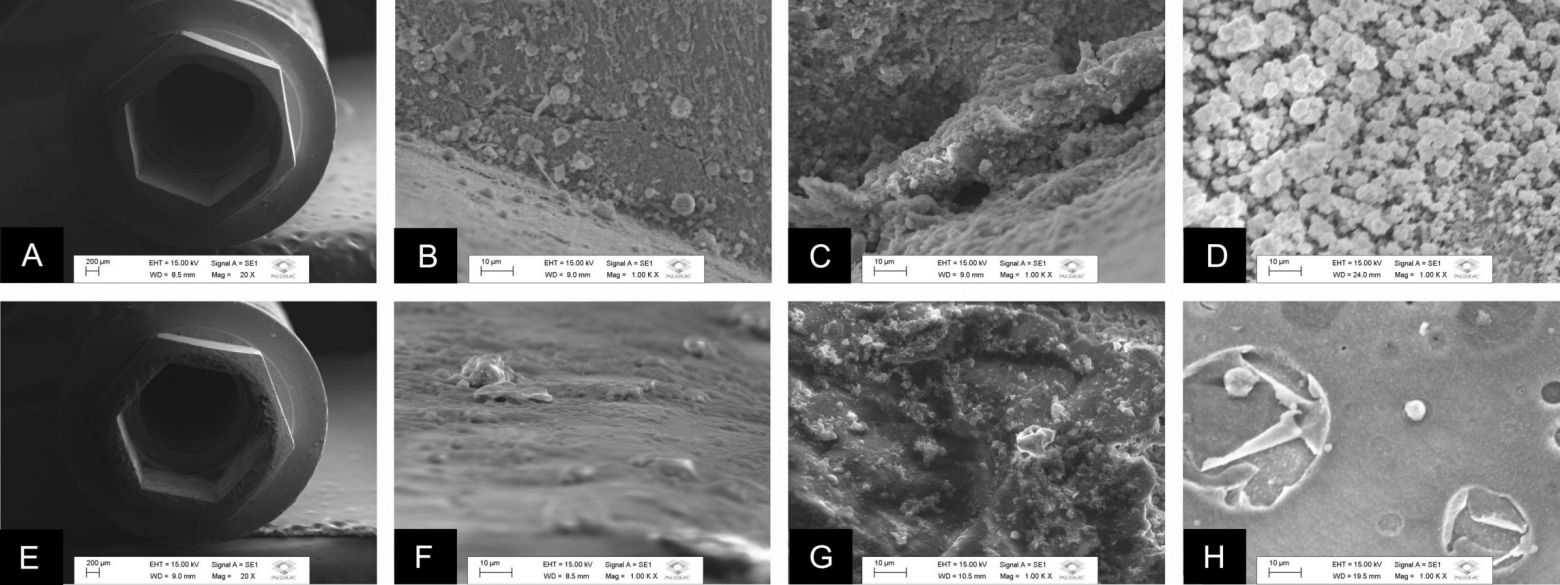
Representative micrographs of the EH specimens after 7 days of incubation with *E*. *faecalis* and *C*. *albicans*, in single or dual-species biofilms, obtained by SEM. A. External region of the EH-C implant; B. HE-C with *E*. *faecalis* in single-species biofilm formed in the internal region of the implant; C. EH-C with *C*. *albicans* in single-species biofilm formed in the internal region of the implant; D. EH-C with dual-species biofilm of *E*. *faecalis* and *C*. *albicans* in the internal region of the implant; E. External region of the EH-T implant with presence of the sealing agent; F. EH-T with presence of colonies of *E*. *faecalis* dispersed on the surface and particles of the sealing agent in the internal region of the implant; G. EH-T with absence of *C*. *albicans* biofilm and presence of particles of the wax agent throughout the surface; H. EH-T with colonies dispersed on the surface in the dual-species biofilm with *E*. *faecalis* and *C*. *albicans*.

Fig 5 shows representative micrographs of the MT specimens after 7 days of incubation with *E*. *faecalis* and *C*. *albicans*, in single or dual-species biofilms, obtained by SEM. In Fig 5A and 5E, images (at 20× magnification) of connection area of the Morse taper (MT) implants from control (MT-C) and test (MT-T) groups, respectively, are presented. On 1000× magnification, Fig 5B shows *E*. *faecalis* in single-species biofilms formed in the MT-C group on the inner surface of the implant, and Fig 5C shows spherical cells of *C*. *albicans* immersed in a large amount of extracellular matrix, confirming the phenotypic characteristic of this type of fungus. Fig 5D shows the dual-species biofilms in the internal surface of the implant in MT-C group. Fig 5F, 5G and 5H under 1000× magnification, are representative of the MT-T group. Fig 5F and 5G represent *E*. *faecalis* and *C*. *albicans* single-species biofilms, respectively, and it is not possible to observe biofilm formation in the internal region of the implant; however, surface irregularities indicate the presence of the sealing agent in this region. Fig 6H is a representative of dual-species biofilm, which shows the same characteristics such as the surface irregularities in the absence of biofilm formation, as described above.

Fig 6 shows representative micrographs of the EH specimens after 7 days of incubation with *E*. *faecalis* and *C*. *albicans*, in single or dual-species biofilms, obtained by SEM. Fig 6A and 6E are representative of the EH-C and EH-T groups, respectively, where the outer portion in the region of the connection can be visualized from the images. Fig 6E shows the presence of surface irregularities on the hexagon, suggesting the presence of the sealing agent in this region, which cannot be observed in the control group (Fig 6A). Fig 6B, 6C and 6D, are representative of the EH-C group for biofilms of *E*. *faecalis*, *C*. *albicans* in single-species, and in dual-species biofilms; a great amount of extracellular matrix and biofilm are observed in these figures, while the Fig 6F, 6G and 6H, which are representative figs of the EH-T group, present only material particles with absence of the biofilm; Fig 6H shows the presence of some spherical cells dispersed on the inner surface of the implant.

**Fig 7:**
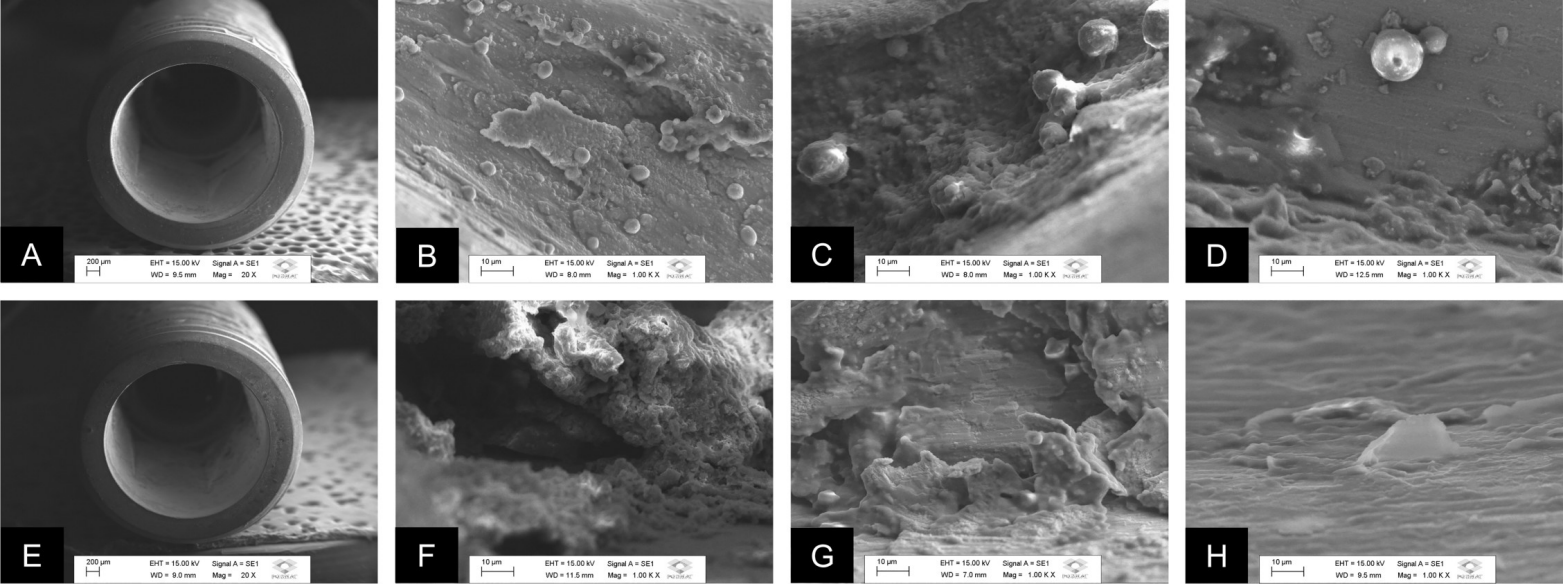
Representative micrographs of the MT specimens after 14 days of incubation with *E*. *faecalis* and *C*. *albicans*, in single or dual-species biofilms, obtained by SEM. A. External region of the MT-C implant; B. MT-C with *E*. *faecalis* in single-species biofilm formed in the internal region of the implant; C. MT-C with C. albicans in single-species biofilm formed in the internal region of the implant; D. MT-C with dual-species biofilm of *E*. *faecalis* and *C*. *albicans* formed in the internal region of the implant; E. External region of the MT-T implant with presence of the agent in the platform; F. MT-T with presence of the sealing agent and some cells of *E*. *faecalis* in single-species biofilm in the internal region of the implant; G. MT-T with absence of *C*. *albicans* in single-species biofilm and presence of particles of the sealing agent near the extracellular matrix in the internal region of the implant; H. CM-T without colonies of *E*. *faecalis* or *C*. *albicans* on the surface of the internal region of the implant and the presence of the agent layer formation.

**Fig 8:**
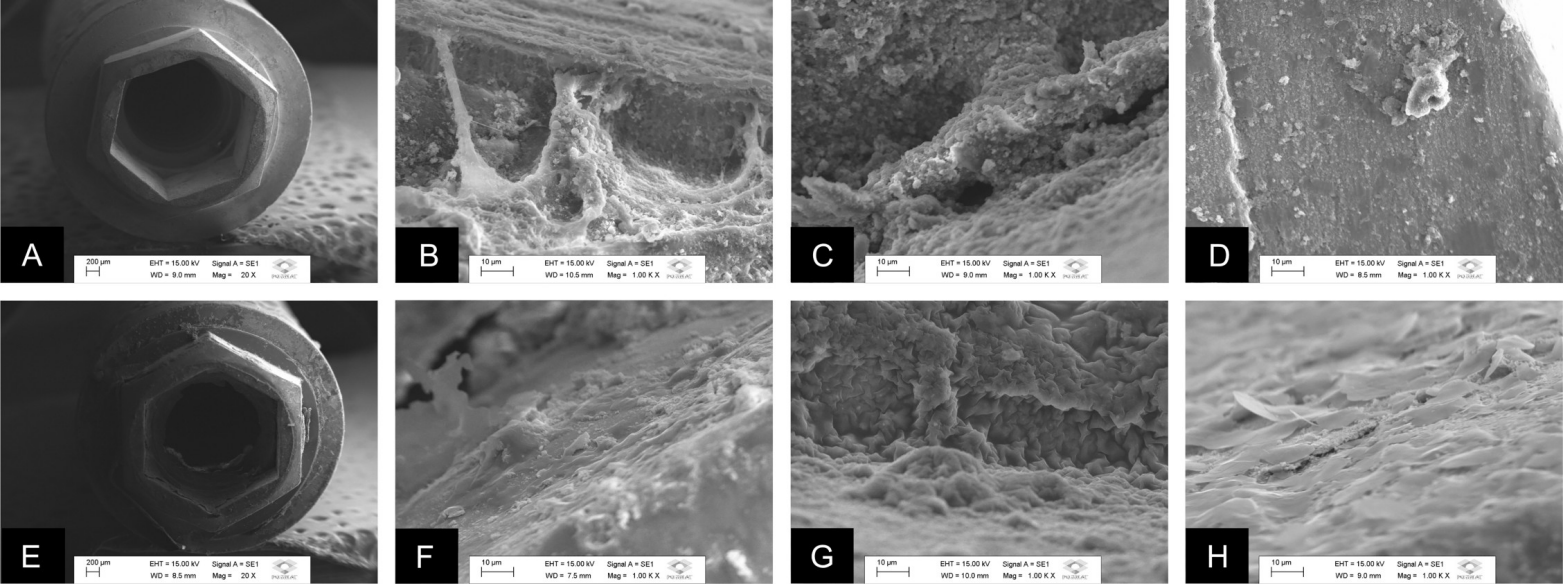
Representative micrographs of the EH specimens after 14 days of incubation with *E*. *faecalis* and *C*. *albicans*, in single or dual-species biofilms, obtained by SEM. A. External region of the EH-C implant; B. EH-C with intense extracellular matrix and biofilm of *E*. *faecalis* formed in the internal region of the implant; C. EH-C with intense extracellular matrix and biofilm of *C*. *albicans* formed in the internal region of the implant; D. EH-C without cells of *E*. *faecalis* and *C*. *albicans* in the internal region of the implant, only particles of the sealing agent; E. External region of the EH-T implant with the presence of the sealing agent in the region of the hexagon and the connection platform; F. EH-T with presence of the sealing agent in the internal region of the implant; G. EH-T with absence of *C*. *albicans* biofilm and presence of the surface-wrapping agent; H. EH-T without colonies of *E*. *faecalis* and *C*. *albicans*, but the presence of the sealing agent.

Figs 7 and 8 shows representative micrographs of the MT and EH specimens, respectively, after 14 days of incubation with *E*. *faecalis* and *C*. *albicans*, in single or dual-species biofilms, obtained by SEM. The presence of the sealing agent is observed in the external region of the MT-T and EH-T (Figs 7E and 8E) in contrast with Figs 7A and 8A from MT-C and EH-C groups in which the presence of the sealing agent is not observed in the same region. The presence of spherical cell agglomerate, which is a phenotypic characteristic of *E*. *faecalis*, is observed in single-species biofilm of MT-C specimen (Fig 7B). Colonies characteristics of *C*. *albicans* were observed in single-species biofilm (Fig 7C) and in dual-species biofilms (Fig 7D) of MT-C groups. Fig 7F shows some cells of *E*. *faecalis* in single-species biofilms. Fig 7G and 7H shows no microbial cells on the specimens of MT-T connections. Fig 8B and 8C shows single biofilms of *E*. *faecalis* and *C*. *albicans*. Dual-species biofilms of *E*. *faecalis* and *C*. *albicans* on EH-C connections was not detected in Fig 8D. The presence of microorganisms organized in biofilms is not observed on EH-T connections (Fig 8F, 8G and 8H); however, the sealing agent is identified on the entire internal surface of the implant, forming a homogeneous film on this surface.

### Visual analysis of bolted joints with and without the sealant

**Fig 9:**
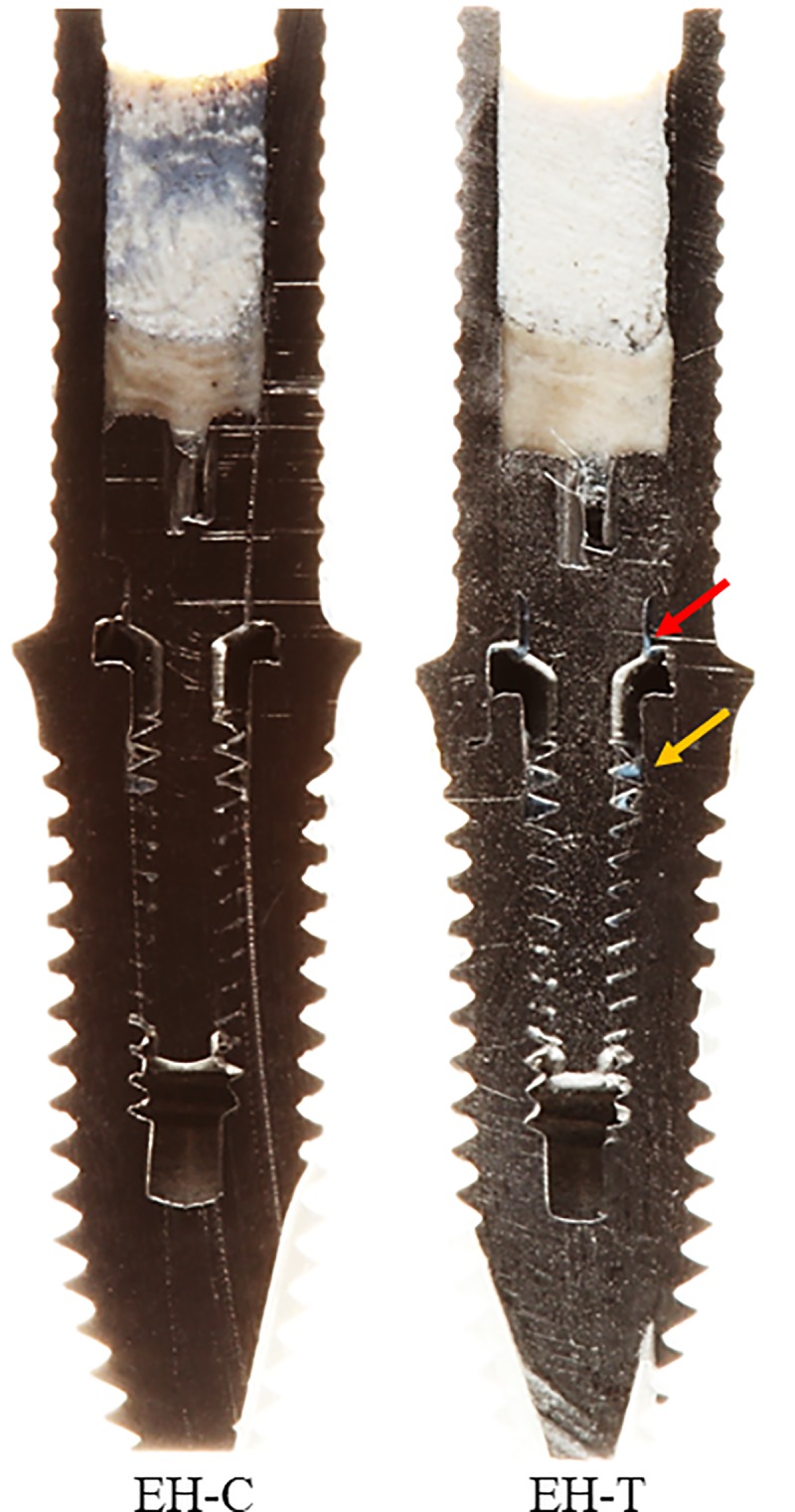
Longitudinally sectioned images obtained from the specimens with MT connection. The red arrow indicates the presence of the sealing agent at the abutment-implant interface, while the yellow arrow points to the sealant between the threads of the retaining screw and the internal thread of the implant at the uppermost portion of the screw joint.

**Fig 10:**
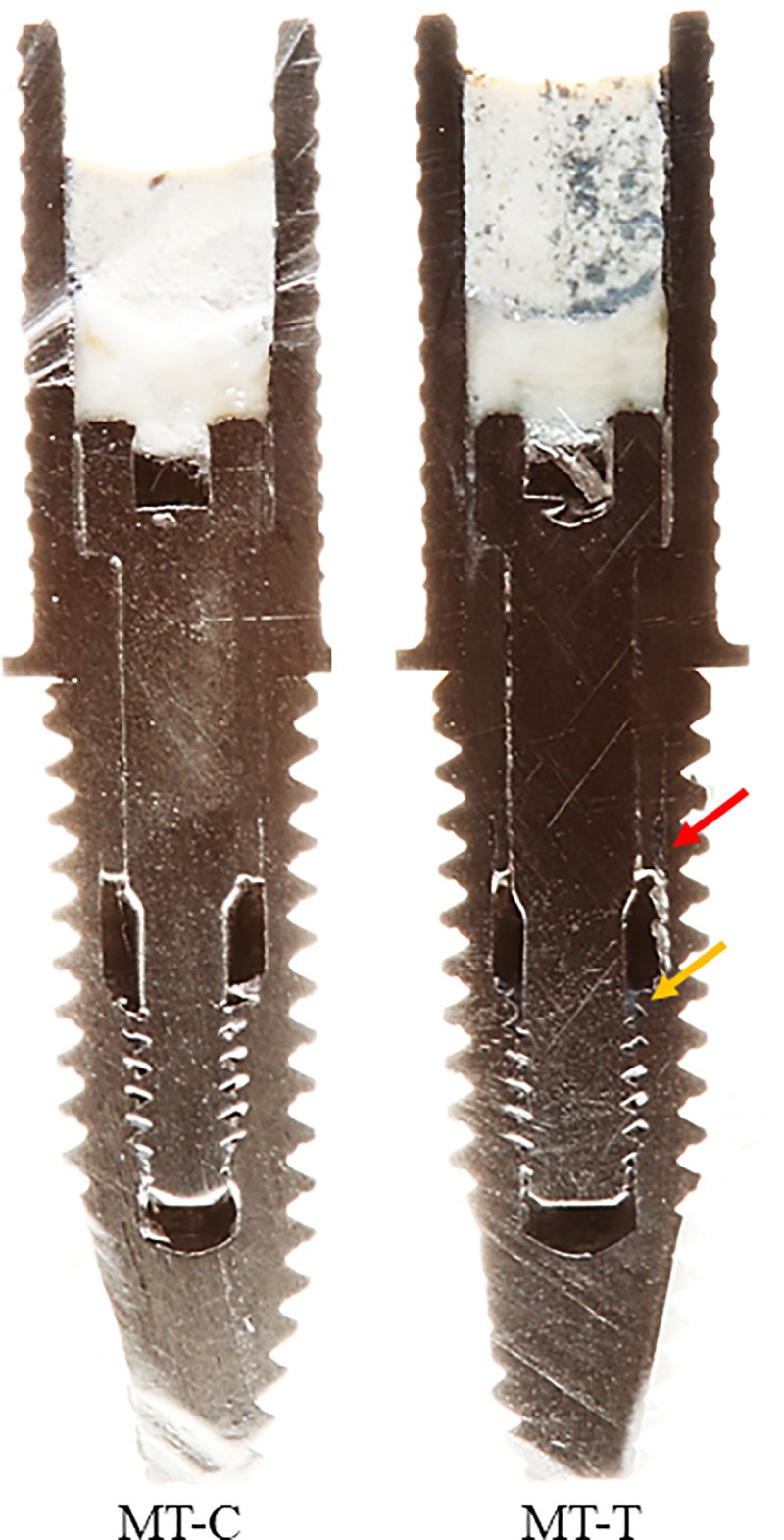
Longitudinally sectioned images obtained from the specimens with EH connection. The red arrow indicates the presence of the sealing agent at the abutment-implant interface, while the yellow arrow points to the sealant between the threads of the retaining screw and the internal thread of the implant at the uppermost portion of the screw joint.

Figs 9 and 10 show the longitudinally sectioned images obtained from the specimens with MT and EH connections, respectively. It was possible to visualize the interior of these connections, the relationship between the structures, and the presence of the sealing agent in the MT-T and EH-T groups. The red arrow indicates the presence of the sealing agent at the abutment-implant interface, while the yellow arrow points to the sealant between the threads of the retaining screw and the internal thread of the implant at the uppermost portion of the screw joint. In the groups with MT-C and EH-T connections, it is possible to verify the absence of material at these interfaces.

## Discussion

Single biofilms of *E*. *faecalis* and *C*. *albicans* infiltrated into the two connections (Morse taper and external hexagon) without any significant difference in the infiltration between either of these connections for the time periods evaluated (7 and 14 days). None of the connections evaluated prevented the infiltration of biofilms when the sealing agent was not used.

However, when the microorganisms (*E*. *faecalis* and *C*. *albicans*) were inoculated together, evident difference was observed in the microbial infiltration between the external hexagon connection groups in relation to the Morse taper connection groups. There was no microbial growth and infiltration into the EH implants, in either the control group or the test group, at 7 or 14 days. It is known that different geometries at the abutment-implant interface can interfere with the penetration of microorganisms into the implants [12]. This result differs from some literature reports [8, 26–30], which assert that the Morse cone type connection is superior to other connections with respect to the susceptibility to microbial infiltration.

However, in a study evaluating water infiltration associated with gentian violet-based dye at the abutment-implant interface with different connections [31], it was observed that the group that received Morse taper implants showed the highest water infiltration, while the groups that received external connection implants had a significantly lower water infiltration. Ricomini Filho (2010) evaluated the infiltration of *Streptococcus sanguinis* inside groups that received implants with external hexagonal connection and Morse taper, and were subjected to mechanical cycling. It was reported that in the Morse taper connection implant group, there was a 67% higher occurrence of bacterial infiltration in comparison to the groups of external hexagonal connection implants, regardless of whether they were subjected to mechanical cycling or not [32]. In the external hexagon group, the presence of bacteria was not observed in any part of the implant or its prosthetic component, which corroborates with the results found in the present study.

This might be due to the fact that the external hexagon on the implant connection platform may have acted as a physical barrier, which possibly prevented the interface microorganisms from penetrating the implant [32]. Furthermore, the fact that the present *in-vitro* study was conducted in a static manner may have made it difficult for the microorganisms to move towards the interior of the implant.

Another aspect to be considered is the difference in the value of the torque applied to the retaining screws of the abutments for HE and CM connections (30 Ncm and 20 Ncm, respectively). These values are, in general, used by several brands of implants. However, the study conducted by Silva-Neto (2012) evaluated the infiltration of high concentration of *Escherichia coli* (0.5 μL, 1.0 μL, and 1.5 μL) in external hexagon implants with screwed abutments using different torque values (10 Ncm, 20 Ncm and 32 Ncm). They observed that in the implants whose abutments were screwed with 32 Ncm torque, none of the specimens showed microbial infiltration. [33] This torque value is close to the value that was used in the present study for the HE groups, and may have been enough to maintain the ideal contact and bonding of the parts.

In contrast, the Morse taper implants showed infiltration and biofilm formation, indicating that even though the torque indicated by the manufacturer was applied, this may not have been enough to seal the interface. This result contradicts previous studies that have demonstrated the effectiveness of this connection in the containment of the microbial infiltration to its interface. [14, 30, 34]

In case of mixed biofilms, microorganisms established interactions that may or may not favor their proliferation and metabolic activity. In dual-species biofilms showed in the present study, CFU counts were similar between *E*. *faecalis* and *Candida albicans* at 14 days, however, there was an increase in the *E*. *faecalis* counts comparing 7 and 14 days, showing bacterial growth. Using a nematode infection model, Garsin and Lorenz (2013) evaluated dual-species biofilms of *C*. *albicans* and *E*. *faecalis* and identified specific interactions occurring between these two pathogens that result in a potentially synergic relationship; a phenomenon in which microorganisms support each other’s growth and proliferation, although a inhibition of hyphal morphogenesis of *C*. *albicans* was observed, attenuating its virulence and avoiding the worm killing (35). Cruz et al. (2013) showed, in a similar model host system for pathogenesis studies with different microbial species, these two important pathogens when together appear to inhibit each other's virulence: not only does *E*. *faecalis* attenuate killing by *C*. *albicans*, but a subsequent exposure to the fungus attenuates *E*. *faecalis* killing as well. They also demonstrated that the presence of *C*. *albicans* appeared to protect *E*. *faecalis* from cell death (36). Comparing 7 and 14 days of dual-species biofilms, there was a significant decrease in the CFU counts of *E*. *faecalis* (MT-T 7d x MT-T 14d), however, both microbial species did not show any growth inside the implants at this time of evaluation. We speculated that dual-species biofilms become thicker and denser outside the implant and the physical presence of the biofilm associated with the sealing agent was enough to completely block the infiltration. Gao et al. (2016) showed that *E*. *faecalis* was more resistant to starvation in coexistence with *C*. *albicans*, *S*. *gordonii*, *A*. *viscosus*, or *L*. *acidophilus*. The dual-species biofilm showed that *E*. *faecalis* formed thicker and denser biofilms on the root canal dentin and glass slides in coexistence with these species (37).

The same microorganisms evaluated in single biofilms presented a different behavior in relation to their ability to infiltrate the abutment-implant interface and to grow inside the implant, in comparison to the mixed inoculation assays. After 7 days of incubation, there was growth of the tested microorganisms only in the control groups (CM-C and HE-C for *C*. *albicans* and *E*. *faecalis*), while the test groups (CM-T and HE-T for *C*. *albicans* and CM-T and HE-T for *E*. *faecalis*) remained free of the formation of biofilms inside the implants. Thus, the effectiveness of the sealing material in preventing microbial infiltration of *E*. *faecalis* and *C*. *albicans* biofilms to abutment-implant interface sealing was evident post 7 days. This result negates the second hypothesis in this study that there is no significant statistical difference in microbial infiltration and formation of biofilms between the test and control groups, regardless of the connection type and the period of incubation.

After 14 days of incubation, microbial infiltration of *E*. *faecalis* to the CM-T group was statistically significant different from its infiltration into the CM-C group. CM-T group was positive for *E*. *faecalis* growth, however, on a smaller scale in comparison to CM-C (CM-T: 5.45 ± 0.49 and CM-C: 0.98 ± 1.95). Thus, there was an effect of the material in containing *E*. *faecalis* infiltration, however, to a lesser extent when compared to the HE-T group. This is justified by what has been already discussed earlier in relation to the different designs and properties of each connection. Thus, it is evident that the efficacy of the material at the abutment-implant interface was important regardless of the incubation period for *E*. *faecalis*. However, no growth of *C*. *albicans* was observed in test groups that received the sealing material, regardless of the type of connection, even after incubating for 14 days, suggesting that the sealing material there was more effective against *C*. *albicans* compared to *E*. *faecalis*.

Jansen and Conrads (1997) have shown that the gap size at the abutment-implant interface varies according to the type of connection and the structural characteristics of the prosthetic abutment. In addition, they found that the mean vertical distance between the components is approximately 0 to 10 μm [35, 36] and the horizontal distance is 60 μm [37, 38]. However, the lack of formation of biofilm in the test groups by *C*. *albicans* is not surprising due to the morphology of the fungus, as *C*. *albicans* are physically large (4 to 6 μm) [39, 40] with hyphae and pseudohyphae, which may have limited their mobility through the gaps into the implants of the test groups, since, in addition to the bonding of the pieces of the screw joint, there was the sealing agent present that may have prevented the entry of the microorganisms inside the implant. [39–41]

This fact can be verified by the photographs (Figs 9 and 10) that show that the sealing agent occupies an important space at the abutment-implant interface and at the screw junction, which acts as a physical barrier, preventing the entry of larger microorganisms such as *C*. *albicans*. The photographic images corroborate with SEM images, which present biofilms in the outermost region of the implant, although growth (CFU/mL) was not observed in the portion that was collected from the implant. Thus, even if gap formation between implant and abutment is unavoidable [20], it is possible to prevent microorganisms from entering the implant chamber, preventing peri-implant diseases.

Upon analysis of the isolated biofilms, a significant difference was observed between the CM-T groups after 7 and 14 days of incubation, with microbial growth of *E*. *faecalis* observed only at 14 days for single-species biofilms. There was also a statistically significant difference between these two groups for mixed cultures of *E*. *faecalis* and *C*. *albicans*, with microbial growth observed only at 7 days. This might be due to the greater thickness of the biofilms formed by mixed culture of *E*. *faecalis* and *C*. *albicans* at 14 days because of which the microorganisms could not cross the abutment-implant interface and penetrate the implant in the presence of the sealing agent, unlike what occurred at 14 days in the isolated cultures of *E*. *faecalis*.

It is also important to note that one of the main advantages of the sealing agent used is its reversibility with screwed prostheses, which makes it possible to loosen the prosthetic part for modifications, repairs, adjustments, and maintenance of screwed prostheses. [19, 42]

A limitation of the study that should be noted is that the study was performed under static conditions, which do not mimic what actually occurs in the oral cavity with variations in temperature and forces that directly and indirectly affect the abutment-implant assembly.

Considering these results and their variability in microbial infiltration depending on isolated or mixed biofilms, it was evident that the use of the sealing agent was effective in containing the microbial infiltration of the tested microorganisms. In this context, important studies are being carried out to test microbial infiltration through connection implants by other microorganisms and their combinations and to perform mechanical analysis, evaluate the properties of this material in relation to its cellular biocompatibility, and to evaluate the tissue response in order to verify the reactions of the organism when in contact with this product, before recommending it for clinical use.

## Conclusion

Based on the results obtained and after considering the limitations of this study, it is possible to conclude that the presence of the sealing agent reduces or eliminate the infiltration of biofilms of *E*. *faecalis* and *C*. *albicans* into the implants, irrespective of the time period of incubation. The external hexagonal connection was more effective than the Morse Taper connection against microbial infiltration for dual-species biofilms, regardless of the time after microbial incubation and the use of the sealing agent.

## Supporting information

S1 Dataset Minimal dataset (Minimal data set.docx). (DOCX)
