# Correction: Sealing agent reduces formation of single and dual-species biofilms of *Candida albicans* and *Enterococcus faecalis* on screw joints at the abutment/implant interface

**DOI:** 10.1371/journal.pone.0229748

**Published:** 2020-03-04

**Authors:** Cecília Alves de Sousa, Jadison Junio Conforte, Karina Sampaio Caiaffa, Cristiane Duque, Wirley Gonçalves Assunção

The first author's initials appear incorrectly in the citation and are indexed incorrectly in PubMed. The correct citation is: Sousa CA, Conforte JJ, Caiaffa KS, Duque C, Assunção WG (2019) Sealing agent reduces formation of single and dual-species biofilms of *Candida albicans* and *Enterococcus faecalis* on screw joints at the abutment/implant interface. PLoS ONE 14(10): e0223148. https://doi.org/10.1371/journal.pone.0223148.

The affiliation for the fifth author is incorrect. Wirley Gonçalves Assunção is not affiliated with #2 but with #1: Department of Dental Materials and Prosthodontic, São Paulo State University (UNESP), School of Dentistry, Araçatuba, São Paulo, Brazil.
