# Supplementary material for: Sealing agent reduces formation of single and dual-species biofilms of Candida albicans and Enterococcus faecalis on screw joints at the abutment/implant interface
Source: PLoS One. 2019 Oct 22;14(10):e0223148. doi: 10.1371/journal.pone.0223148 (PMC6804967; doi:10.1371/journal.pone.0223148)
Supplement: S1 Dataset — (DOCX) [file pone.0223148.s001.docx]

| **Variable-Dependent Variable** | **Goups** | | **Mean Difference** | **Std. Error** |
| --- | --- | --- | --- | --- |
|  |  |  |  |  |
| ***E. faecalis* 7days** | **CM controle** | CM teste | 4,94352* | 0,3029 |
|  |  | HE controle | -0,2650 | 0,3029 |
|  |  | HE teste | 4,94352* | 0,3029 |
|  | **CM teste** | CM controle | -4,94352* | 0,3029 |
|  |  | HE controle | -5,20849* | 0,3029 |
|  |  | HE teste | - | 0,3029 |
|  | **HE controle** | CM controle | 0,2650 | 0,3029 |
|  |  | CM teste | 5,20849* | 0,3029 |
|  |  | HE teste | 5,20849* | 0,3029 |
|  | **HE teste** | CM controle | -4,94352* | 0,3029 |
|  |  | CM teste | - | 0,3029 |
|  |  | HE controle | -5,20849* | 0,3029 |
| ***C. albicans* 7days** | **CM controle** | CM teste | 4,85456* | 0,3558 |
|  |  | HE controle | 0,2685 | 0,3972 |
|  |  | HE teste | 4,85456* | 0,3558 |
|  | **CM teste** | CM controle | -4,85456* | 0,3558 |
|  |  | HE controle | -4,58605* | 0,3302 |
|  |  | HE teste | - | 0,2791 |
|  | **HE controle** | CM controle | - 0,2685 | 0,3972 |
|  |  | CM teste | 4,58605* | 0,3302 |
|  |  | HE teste | 4,58605* | 0,3302 |
|  | **HE teste** | CM controle | -4,85456* | 0,3558 |
|  |  | CM teste | - | 0,2791 |
|  |  | HE controle | -4,58605* | 0,3302 |
| ***E. faecalis* 14days** | **CM controle** | CM teste | 0,85401* | 0,3016 |
|  |  | HE controle | 0,1197 | 0,3016 |
|  |  | HE teste | 5,22930* | 0,2926 |
|  | **CM teste** | CM controle | -0,85401* | 0,3016 |
|  |  | HE controle | -0,7343 | 0,3104 |
|  |  | HE teste | 4,37530* | 0,3016 |
|  | **HE controle** | CM controle | -0,1197 | 0,3016 |
|  |  | CM teste | 0,7343 | 0,3104 |
|  |  | HE teste | 5,10956* | 0,3016 |
|  | **HE teste** | CM controle | -5,22930* | 0,2926 |
|  |  | CM teste | -4,37530* | 0,3016 |
|  |  | HE controle | -5,10956* | 0,3016 |
| ***C. albicans* 14days** | **CM controle** | CM teste | 3,97461* | 0,8090 |
|  |  | HE controle | 0,7630 | 0,8339 |
|  |  | HE teste | 4,70505* | 0,8090 |
|  | **CM teste** | CM controle | -3,97461* | 0,8090 |
|  |  | HE controle | -3,21165* | 0,8339 |
|  |  | HE teste | 0,7304 | 0,8090 |
|  | **HE controle** | CM controle | -0,7630 | 0,8339 |
|  |  | CM teste | 3,21165* | 0,8339 |
|  |  | HE teste | 3,94209* | 0,8339 |
|  | **HE teste** | CM controle | -4,70505* | 0,8090 |
|  |  | CM teste | -0,7304 | 0,8090 |
|  |  | HE controle | -3,94209* | 0,8339 |

| **Values used to build graphs – Single species biofilm** | | | | | | | | | |
| --- | --- | --- | --- | --- | --- | --- | --- | --- | --- |
|  | ***E. faecalis* 7 days** | |  |  |  | ***E. faecalis 14* days** |  |  |  |
|  | CM - C | CM - T | HE - C | HE - T |  | CM - C | CM - T | HE - C | HE - T |
| **Mean difference** | 4,943519 | 0 | 5,208494 | 0 |  | 5,229302 | 4,375296 | 5,109555 | 0 |
| **Std. Error** | 0,70848 | 0 | 1,072215 | 0 |  | 0,71274 | 0,612949 | 0,833705 | 0 |
|  | ***C. albicans* 7 days** | |  |  |  | ***C. albicans 14* days** |  |  |  |
|  | CM - C | CM - T | HE - C | HE - T |  | CM - C | CM - T | HE - C | HE - T |
| **Mean difference** | 4,854562 | 0 | 4,586055 | 0 |  | 5,697812 | 0 | 4,93485 | 0 |
| **Std. Error** | 0,821062 | 0 | 1,228763 | 0 |  | 0,550086 | 0 | 0,099139 | 0 |

**Values behind the means, standard deviations and other measures reported – Single species biofilm**

|  | **Goups** | **Mean** | **Std. Deviation** | **Std. Error Mean** |
| --- | --- | --- | --- | --- |
| Pair 1 | *E. faecalis* 7days –  *E. faecalis* 14days | -0,9973 | 1,0279 | 0,3634 |
| Pair 2 | *C. albicans* 7days - *C. albicans 14days* | -0,1589 | 0,7631 | 0,3413 |

| **Independent Samples Test - Dual species biofilm** | | | | | | |
| --- | --- | --- | --- | --- | --- | --- |
|  |  | **Levene's Test for Equality of Variances** | **t-test for Equality of Means** | | | |
|  |  | **F** | **t** | **Df** | **Mean Difference** | **Std. Error Difference** |
| *E. faecalis C*ontrol group -7days  X  *E. faecalis* Test group - 7days | Equal variances assumed | 7,4396 | 2,8007 | 10,0000 | 0,8818 | 0,3148 |
|  | Equal variances not assumed |  | 2,8007 | 7,4486 | 0,8818 | 0,3148 |
| **Independent Samples Test - Dual species biofilm** | | | | | | |
|  |  | **Levene's Test for Equality of Variances** | **t-test for Equality of Means** | | | |
|  |  | **F** | **t** | **Df** | **Mean Difference** | **Std. Error Difference** |
| *E. faecalis* Control group - 7days  X  *C. albicans* Control group -7days | Equal variances assumed | 2,5922 | -1,3096 | 9,0000 | -0,3559 | 0,2718 |
|  | Equal variances not assumed |  | -1,2555 | 6,5980 | -0,3559 | 0,2835 |
| **Independent Samples Test - Dual species biofilm** | | | | | | |
|  |  | **Levene's Test for Equality of Variances** | **t-test for Equality of Means** | | | |
| *E. faecalis* Control group - 7days  X  *C. albicans* Control group *-* 14days | Equal variances assumed | 4,7052 | -1,6527 | 12,0000 | -0,3895 | 0,2357 |
|  | Equal variances not assumed |  | -1,7359 | 11,9964 | -0,3895 | 0,2244 |

| **Group Statistics – Dual species biofilm** | | | | | |
| --- | --- | --- | --- | --- | --- |
|  | **Dual species groups** | **N** | **Mean** | **Std. Deviation** | **Std. Error Mean** |
| *E. faecalis* 7days | E. faecalis Control group - 7days | 6,0000 | **4,8529** | 0,3512 | 0,1434 |
|  | *E. faecalis* Test group - 7days | 6,0000 | **3,9711** | 0,6866 | 0,2803 |
| **Group Statistics – Dual species biofilm** | | | | | |
|  | **Dual species groups** | **N** | **Mean** | **Std. Deviation** | **Std. Error Mean** |
| *E. faecalis* 7days | *E. faecalis* Control group - 7days | 6,0000 | 4,8529 | 0,3512 | 0,1434 |
|  | *C. albicans* Control group - 7days | 5,0000 | 5,2088 | 0,5469 | 0,2446 |
| **Group Statistics - Dual species biofilm** | | | | | |
|  | **Dual species groups** | **N** | **Mean** | **Std. Deviation** | **Std. Error Mean** |
| *E. faecalis* 7days | *E. faecalis* Control group - 7days | 6,0000 | 4,8529 | 0,3512 | 0,1434 |
|  | *C. albicans* Control group - 14days | 8,0000 | 5,2425 | 0,4883 | 0,1726 |

| **Values used to build graphs – Single species biofilm** | | | | | | | |
| --- | --- | --- | --- | --- | --- | --- | --- |
| ***E. faecalis - 7 days*** | | ***E. faecalis - 14 days*** | | ***C. albicans* - 7 days** | | ***C. albicans* - 14 days** | |
| **MT-C** | **MT-T** | **MT-C** | **MT-T** | **MT-C** | **MT-T** | **MT-C** | **MT-T** |
| 4,5288 | 3,9711 | 5,4584 | - | 5,2088 | - | 5,2425 | - |
| 0,6792 | 0,6866 | 0,4953 | - | 0,5469 | - | 0,4883 | - |
